# Preliminary Evaluation of a Targeted, School-Based Social and Emotional Learning Intervention for at Risk Youth: Football Beyond Borders

**DOI:** 10.1177/0193841X251329459

**Published:** 2025-03-24

**Authors:** Qiqi Cheng, Neil Humphrey

**Affiliations:** 1 Manchester Institute of Education, 57396 University of Manchester

**Keywords:** social-emotional learning (SEL), at-risk youth, mental wellbeing, school, intervention

## Abstract

This brief report provides preliminary independent evidence of the efficacy of Football Beyond Borders (FBB), a targeted, school-based social and emotional learning (SEL) intervention for at-risk youth. FBB includes weekly SEL classroom sessions, activities on the football pitch, 1:1 therapy sessions, holiday support, and rewards trips. Propensity score matching and difference-in-differences estimation were used in a pre-test/post-test control group design to assess the impact of FBB on the mental wellbeing (assessed via the Short Warwick–Edinburgh Mental Wellbeing Scale, SWEMWBS) of participants designated at-risk (*N* = 46 aged 12–14, 78.3% male), passive learners (*N* = 72, aged 12–14, 84.7% male), and role models (*N* = 35, aged 12–14, 85.7% male), with matched control samples derived from a subset of the #BeeWell cohort study (*N* = 8015). A significant intervention effect was observed for at-risk youth, with FBB leading to an increase of approximately 2.4 SWEMWBS points (*d* = 0.44). No significant intervention effects were observed for passive learners or role models. These results indicate that FBB can improve the mental wellbeing of at-risk youth. Accordingly, an explanatory trial is warranted.

## Introduction

This study focuses on a novel approach to improving adolescent mental wellbeing, by which we mean feeling good (hedonia) and functioning well (eudaimonia) (Deighton, 2016). The context for the research is a major public health crisis that has unfolded over the last two decades (Humphrey, 2021), during which we have seen youth wellbeing decline (Marquez & Long, 2021) and the prevalence of mental health difficulties increase significantly (NHS Digital, 2022). More generally, adolescence is a sensitive period of rapid development and a time of increased vulnerability for the onset of lifetime cases of mental health problems, which peaks between the ages of 14 and 15 years (Solmi et al., 2022). Adolescent mental health and wellbeing is of vital importance given its predictive utility in terms of adult physical, labour market/socioeconomic, and relational outcomes (Goodman et al., 2015). Accordingly, growing the intervention evidence base in this area is a research priority (Mei et al., 2020).

Schools are central sites of mental health promotion for at-risk youth (i.e. those experiencing a range of circumstances that make increase their vulnerability to the onset, maintenance, or escalation of social, emotional, behavioural and/or academic difficulties). A recent meta-analysis of school-based mental health interventions revealed meaningful effects, supporting their use as part of a holistic, multi-tiered approach to prevention (*d* = 0.42) (Zhang et al., 2023). However, numerous moderators were identified, with larger effects for selective/targeted approaches (vs universal); those implemented in secondary school settings (vs primary school); and, those implemented by an external professional (vs teacher) (Zhang et al., 2023). Furthermore, there is evidence of a ‘developer effect’ for some interventions for at-risk youth, with larger effects evident when researchers have been involved in intervention design/delivery, reinforcing the need for independent evaluation (Valdebenito et al., 2019). The current study brings these elements together, providing the first independent evidence of the efficacy of a novel targeted intervention, implemented in secondary school settings by an external professional: Football Beyond Borders (FBB).

Building the evidence base for programmes like FBB addresses a major problem, which is that schools overwhelmingly use interventions that have little or no empirical validation (Nisar et al., 2024). This reflects a stark reality for the providers of such interventions: robust, independent evaluation is prohibitively expensive and inaccessible to most (Molloy, 2019). In a broader context in which spending on early intervention services for young people has declined by 44% since 2010 (Larkham, 2024), it is vital that increasingly scarce financial resources are allocated judiciously to support the implementation of programmes that can make a real difference to the most vulnerable. The study described herein forms part of a broader programme of research to address this issue, in which we are working with intervention providers to provide pragmatic but rigorous estimates of efficacy by harnessing the power of large-scale universal wellbeing measurement programmes such as the #BeeWell initiative (#BeeWell Research Team, 2021). Funders and commissioners can use the evidence generated to guide decision-making about the best use of their resources (Franklin et al., 2024).

### Football Beyond Borders (FBB)

FBB is a targeted, school-based social and emotional learning (SEL) intervention. Youth recruited to participate are considered *at-risk* by their school, denoted by evidence of significant behavioural issues; a history of fixed-term or permanent exclusions; significant socio-economic disadvantage; exposure to multiple adverse childhood experiences; and/or, identified additional needs. Young people meeting one or more of these criteria are recruited to work alongside those considered to be *passive learners* (predicted not to meet national expectations in English and Maths at the end of secondary school; emerging behavioural issues; low homework completion rates; and/or special educational needs) and *role models* (top 20% academic achievement at entry to secondary school; play for school football team; and/or predicted to exceed national expectations in English and Maths at the end of secondary school) during the 38-week intervention.

FBB comprises four key elements: (i) weekly project-based classroom SEL sessions, delivered alongside weekly pitch-based football activities by a qualified youth practitioner; (ii) weekly 1:1 therapy sessions for those requiring additional support led by a qualified counsellor; (iii) regular engagement with parents/carers to build a picture of home life, keep them updated on their child’s FBB progress, and identify ways in which they can be better supported; and, (iv) reward trips, including visits, residentials, and work experience opportunities, involving inspirational figures from the world of football and media. The FBB theory of change suggests that these inputs improve key intermediate outcomes (e.g. social and emotional skills, mental wellbeing, behaviour, and attendance) via mechanisms of change that include the development of consistent and long-term relationships, a sense of belonging, and an engaging and relatable curriculum (Football Beyond Borders, 2022).

Our focus in the current study is the impact of FBB on mental wellbeing. This was driven by theory (i.e. improved mental wellbeing is a key theorised outcome of FBB participation); pragmatism (i.e. the availability of a mental wellbeing measure that is common to both the FBB and #BeeWell datasets); and, empirical evidence (i.e. targeted school-based programmes have previously been shown to improve mental health outcomes for at-risk youth; Zhang et al., 2023).

### State of the Art

The current study provides important new evidence pertaining to the efficacy of FBB. The charity has seen significant growth and reach since its formation a decade ago – in any given school year, 2000 young people in the UK are enrolled (Football Beyond Borders, 2022). It is imperative that interventions like FBB are subject to independent evaluation, to ‘better find out what works, what doesn’t, what is a poor use of funds and what interventions harm children’s wellbeing’ (Franklin et al., 2024, p.22). The FBB intervention model is novel in its combination of the distinct elements highlighted above, but we particularly highlight the potential utility of building a relationship with a trusted adult (in this case, a youth practitioner) as a means through which to support the wellbeing of those at-risk (Frederick et al., 2023), and something that is lacking for a significant proportion of the youth population (Ashton et al., 2021). Building such a relationship both in the classroom and on the football pitch, with SEL providing a skills-based foundation to help young people navigate challenging situations more effectively, FBB has the potential to be a game-changer (pun fully intended) for some of our most vulnerable adolescents.

The objective of the current study was therefore to provide preliminary independent evidence of the efficacy of FBB in improving the mental wellbeing of at-risk youth. Thus, our principal research question is, ‘Does participation in FBB increase mental wellbeing among at-risk adolescents?’ Based on the intervention’s theory of change (Football Beyond Borders, 2022), and the presence of empirically verified effect moderators noted earlier (Zhang et al., 2023), we hypothesized that *exposure to FBB would lead to significant improvements in mental wellbeing of at-risk youth, when compared to a propensity score matched control group of unexposed participants*. No specific predictions were made in relation to intervention effects for passive learners and role models, though we include exploratory analyses for these groups which serve as a robustness check for our focal analyses pertaining to at-risk youth (i.e. these effectively served as internal control groups, given the primary emphasis of FBB on the at-risk group). The analyses reported herein (analysis code available on request) provide the first independent evidence regarding the efficacy of FBB, a crucial consideration given the existence of developer effects for analogous interventions (Valdebenito et al., 2019).

## Methodology

### Design

A pre-test-post-test difference-in-differences design was used. FBB participants completed the SWEMWBS measure at the beginning of the intervention (T0), and at 12-month follow-up (T1). These anonymised data were shared with the authors, who derived a control group from the #BeeWell study’s longitudinal cohort (#BeeWell Research Team, 2021), who complete measures every 12 months, meaning that the measurement lag was equivalent across groups. The process through which said control group were derived is outlined in the subsections that follow. The study received ethical approval from the University Research Ethics Committee (UREC) at the University of Manchester (ref number: 2023-16863–31046). This study was not preregistered.

#### Causal Inference Through Difference-In-Differences Estimation and Propensity Score Matching

Randomised trials are rightly considered to be the optimal means through which to determine the efficacy of an intervention. However, they are very costly endeavours and are often infeasible or impractical for other reasons (Benedetto et al., 2018). In such circumstances, a variety of other options are available that permit causal inference regarding intervention effects, of which two are pertinent in the current study: Difference-In-Differences (DiD) estimation and propensity score matching (PSM).

**Figure 1.**
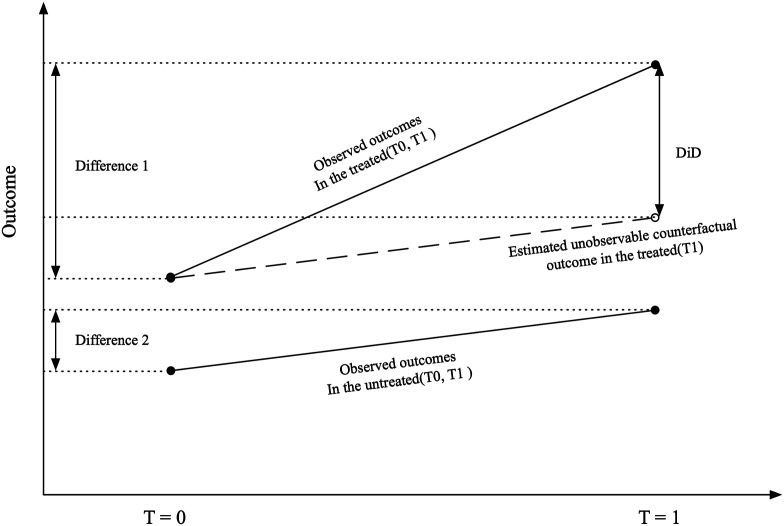
Stylized Exposition of Identification in the DiD model.

Using a quasi-experimental approach, DiD is one of the most commonly used methods in impact evaluation. In a canonical example, one group is exposed to an intervention and the other is not, with measurements taken before (pre-test) and after the intervention (post-test). An intuitive idea is to directly compare the difference between the outcome of interest before and after the intervention. However, the difference between the post-test and the pre-test incorporates not only the hypothesized intervention effect but also the effect of time trends, and other possible effects. Therefore, DiD uses the difference between the post-test and the pre-test for the control group to estimate the effects of other factors (the effects of unobserved variables). Under the parallel trend assumption, the intervention effect is obtained by subtracting the change in the intervention group (difference 1) from the change in the control group (difference 2) (Fredriksson & Oliveira, 2019; Schwerdt & Woessmann, 2020) (see Figure 1).

Our DiD specification takes the following canonical two-way fixed effects (TWFE) regression specification

$$y_{i,t}=\alpha_{i}+\phi_{t}+{\beta \cdot D}_{i,t}+\epsilon_{i,t}$$

The dependent variable $y_{i,t}$ is the wellbeing outcome of individual i measured at time *t.* Our model includes individual fixed effect $\alpha_{i}$ , time fixed effect $\phi_{t}$ and the indicator of treatment status of individual i previous to time *t,* $D_{i,t}$ . For brevity, we have defined $D_{i,t}={FBB}_{i}\times {Post}_{t}$ , where ${FBB}_{i}$ is a binary variable indicating whether individual i is in the FBB program, and ${Post}_{t}$ is a binary variable indicating the post-intervention period. The estimated $\hat{\beta }$ is the coefficient of interest, corresponding with the average treatment effect on the treated (ATT) under the parallel trends and no anticipation assumptions.

Although DiD is considered to be a rigorous approach to control the effect of time-invariant confounders, a concern is that intervention and control groups may differ in ways that would affect the longitudinal trends in their outcomes, thereby undermining the parallel trends assumption. It is here where PSM can be used to handle this type of confounding and produce an unbiased effect estimate (Stuart et al., 2014). In the approach to PSM used in the current study, each participant in the intervention group is matched to a control group participant using their respective propensity scores. The propensity score is the probability of receiving the intervention, conditional on a set of confounders (e.g. pre-test outcome, plus relevant socio-demographic characteristics), estimated via logistic regression (Benedetto et al., 2018).

### Participants

Intervention group data pertaining to 46 at-risk youth, 72 passive learners, and 35 role models drawn from a convenience sample of 13 secondary schools were securely shared with the study authors by FBB. The focal at-risk group were 78% male, 50% eligible for free school meals (FSM), 35% identified as having special educational needs (SEN), and 76% were in Year 8 (aged 12/13) when they participated in FBB. Control group data were drawn from a subset of the #BeeWell longitudinal cohort dataset (BeeWell Research Team, 2021) whose records contained sufficient relevant data to enable them to be considered in the PSM process (*N* = 8015 drawn from 104 secondary schools; final matched control group sample drawn from 38 secondary schools). Prior to matching, 50% were male, 21% were eligible for free school meals (FSM), 12% identified as having special educational needs (SEN), and 99% were in Year 8 (aged 12/13). Both datasets used in the current study were drawn from Greater Manchester, England, with data being generated between 2021 and 2023.

### Measures

#### Mental Wellbeing

The self-report Short Warwick Edinburgh Mental Wellbeing Scale (SWEMWBS) comprises seven items (e.g. ‘I’ve been feeling optimistic about the future’), each of which are endorsed on a five-point scale (none of the time, rarely, some of the time, often, all of the time). The measure has favourable psychometric characteristics, including strong internal consistency, convergent validity, construct validity, and discriminant validity (Ringdal et al., 2018). Furthermore, it was recently identified as an optimal youth wellbeing measure in a comprehensive analysis of spanning assessment of dimensionality, measurement invariance, and convergent validity across nine different instruments available in the #BeeWell dataset (e.g. *Me and My Feelings Survey*, *Perceived Stress Scale*, and *Positive and Negative Affect Schedule*) (Black et al., 2024). Finally, it has also been successfully mapped to Office for National Statistics life satisfaction scores, enabling changes in SWEMWBS scores to be converted to wellbeing years (so called ‘WELLBYS’) for economic analysis (Franklin, 2024). Thus, it has significant advantages over other available measures in terms of its brevity, psychometric properties, and utility. Critically, SWEMWBS was also common to both datasets used in the current study (see *Design* above), enabling the DiD/PSM procedures outlined earlier to be undertaken.

Possible scores on SWEMWBS range from 7 to 35, with higher scores indicating better mental wellbeing. Internal consistency for the scale in the current study was high (intervention group α = 0.79 at T0 and 0.85 at T1; control group α = 0.85 at T0 and 0.88 at T1).

#### Covariates

Participant gender (male/female), FSM eligibility (yes/no), SEN (yes/no), and year group (7/8/9; dummy per year group) drawn from school records were included in the FBB dataset alongside pre-test and post-test outcome scores. In the #BeeWell dataset, equivalent indicators were derived from linked administrative data provided by Local Authorities. Two further covariates were used to facilitate the matching process. First, #BeeWell survey responses to an item asking about happiness with attainment (#BeeWell Research Team, 2021) were used to mimic the school nomination process regarding the academic attainment selection criterion used by FBB. Second, the #BeeWell adversity index (Marquez et al., 2023) was used to mimic the exposure to school nomination process regarding the adverse childhood experiences selection criterion used by FBB.

## Results

### Propensity Score Matching

**Figure 2.**
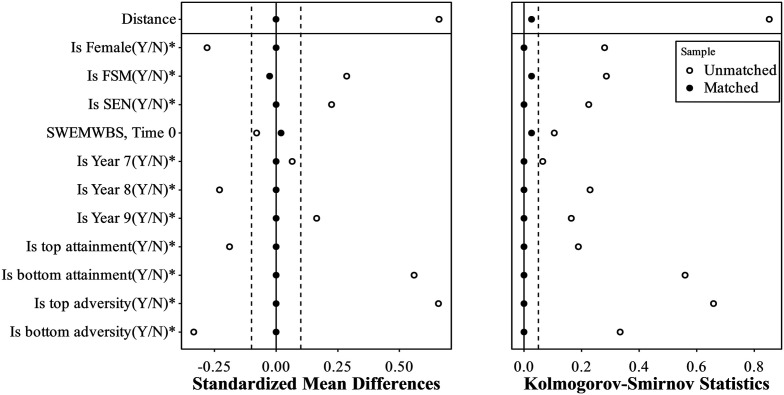
Covariate Balance Before and After Propensity Score Matching. Note. The dash lines show a 0.1 threshold for the SMD and a 0.05 threshold for the KS statistics. * indicates variables for which the displayed value is the raw (unstandardized) difference in means.

**Figure 3.**
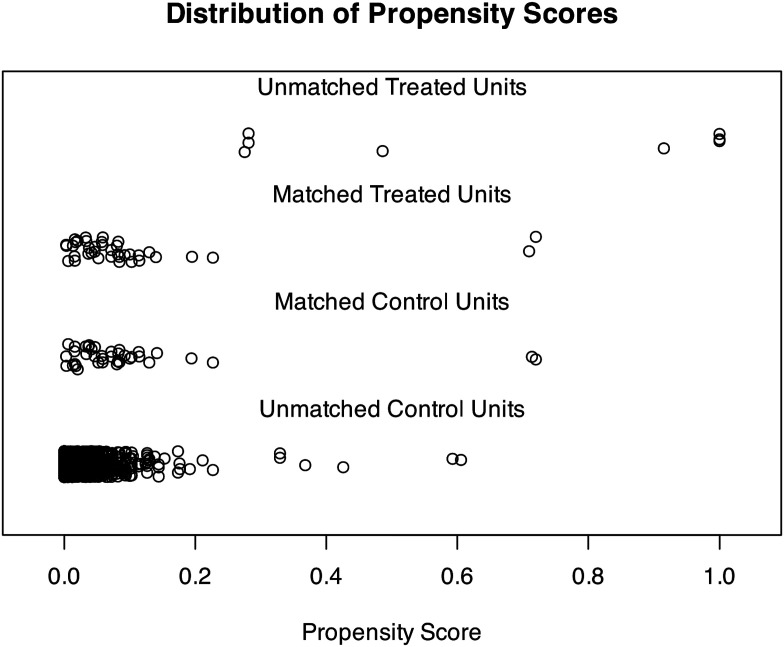
Jitter Plot Demonstrating the distribution of propensity scores in matched and unmatched samples.

A nearest neighbour matching algorithm was used and yielded 38 matched pairs of at-risk youth across the intervention and control groups (*N* = 76 total), meaning 8/46 cases from the FBB dataset and 7977/8015 cases from the #Beewell dataset were not matched. Propensity scores were generated based on pre-test outcome score, gender, FSM eligibility, year group, happiness with attainment, and adversity index. Matching dramatically reduced the distance of the propensity score between the FBB and control groups (see Table 1, and Figures 2 and 3), with standardized mean differences dropping from 0.66 to 0 (below the 0.1 threshold), the Kolmogorov-Smirnov test statistic dropping from 0.85 to 0.03 (below the 0.05 threshold), and the overlap coefficient dropping from 0.46 to 0 (below the 0.1 threshold) (Franklin et al., 2014). In sum, the application of PSM enabled the derivation of a well-matched control group to be used in DiD estimation.

**Table 1.** Group Characteristics before and after Propensity Score Matching.

		Unmatched				Matched			
		FBB = 0 (*N* = 8015)	FBB(AR) = 1 (*N* = 46)	p value	SMD	FBB = 0 (*N* = 38)	FBB(AR) = 1 (*N* = 38)	p value	SMD
SWEMWBS T1 SWEMWBS T0		23.43 (5.82)	23.17 (5.26)	0.765	0.046	20.89 (5.89)	23.39 (5.11)	0.052	0.453
		23.61 (5.69)	23.20 (5.27)	0.62	0.076	23.29 (5.17)	23.39 (5.40)	0.931	0.02
Is SEN (%)	0	7031 (87.7)	30 (65.2)	<0.001	0.55	23 (60.5)	23 (60.5)	1	<0.001
	1	984 (12.3)	16 (34.8)			15 (39.5)	15 (39.5)		
Is female (%)	0	4027 (50.2)	36 (78.3)	<0.001	0.611	31 (81.6)	31 (81.6)	1	<0.001
	1	3988 (49.8)	10 (21.7)			7 (18.4)	7 (18.4)		
Is FSM (%)	0	6303 (78.6)	23 (50)	<0.001	0.626	20 (52.6)	21 (55.3)	1	0.053
	1	1712 (21.4)	23 (50)			18 (47.4)	17 (44.7)		
Is year 7 (%)	0	8015 (100)	43 (93.5)	<0.001	0.374	38 (100)	38 (100)	NA	<0.001
	1	0 (0)	3 (6.5)			0 (0)	0 (0)		
Is year 8 (%)	0	76 (0.9)	11 (23.9)	<0.001	0.742	3 (7.9)	3 (7.9)	1	<0.001
	1	7939 (99.1)	35 (76.1)			35 (92.1)	35 (92.1)		
Is year 9 (%)	0	7939 (99.1)	38 (82.6)	<0.001	0.594	35 (92.1)	35 (92.1)	1	<0.001
	1	76 (0.9)	8 (17.4)			3 (7.9)	3 (7.9)		
Is top attainment (%)	0	6500 (81.1)	46 (100)	0.002	0.683	38 (100)	38 (100)	NA	<0.001
	1	1515 (18.9)	0 (0.0)			0 (0)	0 (0)		
Is bottom attainment (%)	0	4488 (56)	0 (0.0)	<0.001	1.595	0 (0)	0 (0)	NA	<0.001
	1	3527 (44)	46 (100)			38 (100)	38 (100)		
Is top adversity (%)	0	5280 (65.9)	0 (0.0)	<0.001	1.965	0 (0)	0 (0)	NA	<0.001
	1	2735 (34.1)	46 (100)			38 (100)	38 (100)		
Is bottom adversity (%)	0	5336 (66.6)	46 (100)	<0.001	1.002	38 (100)	38 (100)	NA	<0.001
	1	2679 (33.4)	0 (0)			0 (0)	0 (0)		

*Note.* For binary variables, *p*-values are obtained from Pearson’s chi-squared test with continuity correction and SMD (standardized mean difference) is the multivariate Mahalanobis distance between group-specific proportions. For continuous variables, *p*-values are obtained from one-way ANOVA assuming equal variance. The statistics are obtained using the R package *tableone*. SWEMWBS = Short Warwick Edinburgh Mental Wellbeing Scale score. SEN = Special educational needs. FSM = Free school meals eligibility. AR = At risk.

### The Impact of FBB on the Mental Wellbeing of At-Risk Youth

The results from the DiD estimator with the matched data are reported in Table 2. The effect of FBB on SWEMWBS scores is statistically significant and positive, at about 2.39 points (95% CI = [0.25, 4.54]), equivalent to *d* = 0.44. This effect is stable after we control for the interaction between the post-intervention period (time) and the covariates noted earlier.

**Table 2.** The Impact of FBB on Mental Wellbeing (SWEMWBS).

	Model 1	Model 2
*ATT* $D_{i,t}$	2.39 (0.25, 4.54)	2.36 (0.16, 4.55)
Time FE $\phi_{t}$	Yes	Yes
Individual FE $\alpha_{i}$	Yes	Yes
$X_{i}\times {Post}_{t}$	No	Yes

*Note.* The table reports the DiD effect resulting from employing the two-way fixed effects (TWFE) regressions with *fixest* package for R (Berge L, 2018).

Both models included the time fixed effect and individual fixed effect. In model 2 we controlled for the interaction between post treatment period and covariates, $X_{i}\times {Post}_{t}$ . The covariates include SEN, Gender, FSM, Year 8, Year 9. Year 7, top attainment, bottom attainment, top adversity and bottom adversity indicators were eventually dropped as there was no variance in these variables in the matched sample (see Table 1).

The 95% confidential intervals of the estimate are shown in parentheses.

SWEMWBS = Short Warwick Edinburgh Mental Wellbeing Scale score. SEN = Special educational needs. FSM = Free school meals eligibility. AR = At risk. ATT = Average treatment effect on the treated (over the region of common support). $D_{i,t}$ is the indicator of treatment status of individual i previous to time *t.*

### Robustness and Sensitivity Checks

Applying the same methods described above to FBB passive learners and role models as placebo tests, we found that the effect of FBB on the mental wellbeing of passive learners to be positive but not statistically significant, at about 0.36 points (95% CI = [−1.50, 2.21]), equivalent to *d* = 0.07. Similarly, the effect of FBB on the mental wellbeing of role models was also positive but not statistically significant, at about 0.39 points (95% CI = [−2.76, 3.54]), equivalent to *d* = 0.09 (see Supplemental Materials).

**Figure 4.**
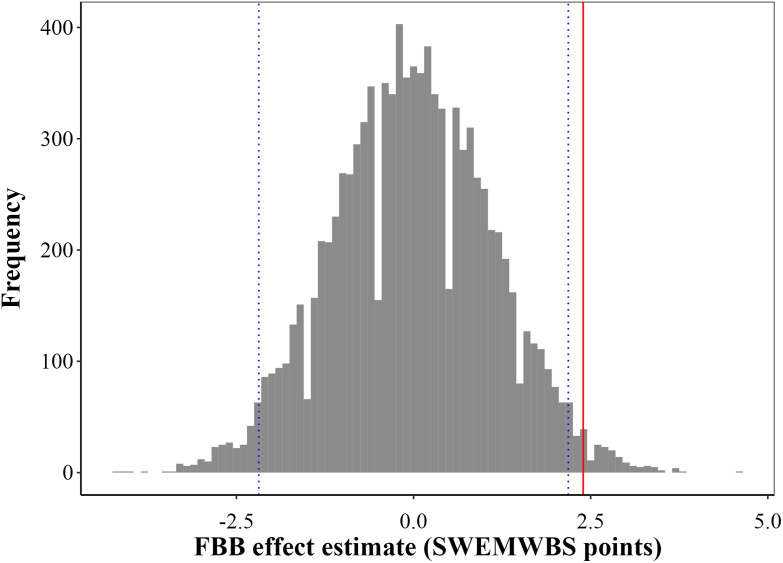
Distribution of Coefficients in the Falsification Permutation Test. Note. The graph illustrates the empirical density of estimated coefficients obtained from 10000 simulations wherein the FBB status was randomly assigned to participants within the matched data set. The red solid vertical line represents the effect size reported in Model 1 in Table 2. The dashed blue line represents the 95% CI of the empirical distribution in this falsification test.

We also conducted a permutation test by randomly assigning FBB group status (FBB = 1) to participants to determine the influence of any unaccounted variables on the study results. This methodology simulated a scenario where the intervention was implemented randomly (La Ferrara et al., 2012). Since the ‘fake’ FBB = 1 variable is generated randomly, it should theoretically produce a non-significant estimate close to zero. If the DiD estimate was statistically significant, it would indicate that it had been mis-specified. This test was repeated 10, 000 times in order to enhance its power. The distribution of estimates from the 10, 000 runs is illustrated in Figure 4 alongside the benchmark estimate of 2.39. Using a randomly constructed FBB = 1 variable, the estimates are clearly centred around zero with a standard deviation of 1.13, suggesting no effect. The benchmark estimate, in contrast, lies outside the 95% empirical interval (−2.24, 2.18) of the entire distribution. Collectively, our findings indicate that the positive and significant effect of the FBB on the mental wellbeing of at-risk youth is not due to unobserved factors.

In addition, a series of sensitivity analyses were performed, involving multiple imputation of missing data; use of transformed SWEMWBS scores; use of z-transformed SWEMWBS scores; use of alternative DiD estimator; alternative matching approaches (e.g. nearest neighbours without a specified caliper width, exact, and genetic); and, combinations of these (e.g. multiple imputation plus use of transformed SWEMWBS scores) (see Supplemental Materials). The intervention effect identified in the main analysis was largely insensitive to researcher-led analytic decisions, reinforcing the robustness of our findings. Accordingly, our study hypothesis was supported.

## Discussion

The objective of the current study was to provide preliminary independent evidence of the efficacy of Football Beyond Borders (FBB), a targeted, school-based social and emotional learning (SEL) intervention. Using difference-in-differences (DiD) estimation and propensity score matching (PSM) in the absence of random allocation, the impact of FBB on the mental wellbeing of at-risk youth was assessed, and a statistically significant, positive intervention effect was identified. No significant intervention effects were observed for passive learners or role model FBB participants.

### Implications and Future Directions

The intervention effect size observed in the current study 2.39 SWEMWBS points (equivalent to *d* = 0.44, or a 17-percentile improvement) is likely practically significant, being within the range noted as meeting thresholds for statistically important change in a responsiveness evaluation with a clinical sample (Shah et al., 2018). It also compares favourably to the average effect size for school-based, targeted mental health interventions reported in a recent meta-analysis (*d* = 0.42; Zhang et al., 2023). Collectively, we can infer that FBB is making a meaningful difference to the mental wellbeing of at-risk youth. Helpfully, a social return on investment analysis undertaken in parallel to the research reported herein indicated that the programme also generates £2.20 in monetized wellbeing benefits for every £1 invested (Franklin, 2024). Given the well-documented public health crisis in this area (Humphrey, 2021) and the prevalence of school-based interventions for which there is little or no supporting empirical validation (Nisar et al., 2024), these findings provide important new evidence that can help funders and commissioners make informed decisions about the best use of their resources (Franklin et al., 2024).

However, our analysis provides only preliminary evidence for FBB, and several next steps are recommended. First, rigorous evidence pertaining to other theorised outcomes of FBB (e.g. improved social and emotional skills, behaviour, attainment and attendance; Football Beyond Borders, 2022) needs to be gathered. Second, analysis of treatment effect moderators (e.g. intervention compliance, contextual and environmental factors) is needed to better understand how and why, and under what circumstances FBB works. Third, longer-term maintenance of the positive effects reported here needs to be established. Fourth, analysis how the different constituent components of the FBB program individually contribute to improved mental well-being could provide crucial insights for program optimization and replication. This analysis would not only enhance our understanding of the intervention’s mechanisms, but also inform evidence-based improvements to the programme design. Collectively, these steps could be undertaken together in the context of an independent, explanatory randomised trial.

### Strengths and Limitations

The current study had numerous strengths, including the use of DiD, PSM, robustness and sensitivity checks (i.e. placebo and permutation tests), and the fact that both the intervention and control samples were drawn from the same general population and time period. However, there are also limitations that need to be borne in mind. First, the available FBB samples were relatively small and drawn from a convenience sample, limiting statistical power and generalisability. This issue was amplified by wastage ensuing from the PSM process (i.e. some unmatched participants in each analysis, leading to their exclusion). While this was ultimately inconsequential for the focal analysis, in which a statistically significant intervention effect was observed, it may have yielded consequences for the analyses pertaining to passive learners and role models, for whom one might reasonably expect a much smaller intervention effect, requiring a considerably larger sample than was available in order to be detectable. This limitation could be overcome in a fully powered explanatory trial. Second, although the intervention effect identified in the main analysis was largely insensitive to researcher-led analytic decisions (e.g. multiple imputation; use of transformed SWEMWBS scores; use of z-transformed SWEMWBS scores; use of alternative estimator; seven out of the nine alternative matching approaches), there were some exceptions (i.e. two of the nine alternative matching approaches did not produce significant intervention effects, although one of these was *p* = .05), reinforcing the need for caution and effect replication. Third, although the groups were well matched on multiple key criteria (e.g. pre-test outcome score, gender, FSM eligibility, SEN, age, attainment, and adversity), it is important to note other variables that could influence longitudinal trends in wellbeing that were not included in the matching process (e.g. family characteristics, such as parental education, household income, family structure, or parental involvement, which likely influence both programme participation and youth outcomes). However, the study was subject to natural limits, including the relatively small sample size (i.e. we were mindful of the need to avoid overfitting), and a pragmatic matching process (i.e. matching was contingent on data availability). Fourth, close proxies were used for two of the seven matching variables (attainment and adverse childhood experiences; although as above, this was a pragmatic decision that enabled the derivation of a more analogous control sample than if these variables had been excluded). Finally, use of a DiD design involving multiple pre- and post-test measurements would have been preferable as it could have provided more robust evidence to support the parallel trends assumption than was possible in our simple pre-test-post-test design involving only two data points. Despite these limitations, the current study provides robust preliminary evidence that FBB improves the mental wellbeing of at-risk youth. Accordingly, an explanatory trial is warranted.

## Supplemental Material

Supplemental Material - Preliminary Evaluation of a Targeted, School-Based Social and Emotional Learning Intervention for at Risk Youth: Football Beyond Borders Supplemental Material for Preliminary Evaluation of a Targeted, School-Based Social and Emotional Learning Intervention for at Risk Youth: Football Beyond Borders by Qiqi Cheng and Neil Humphrey in Evaluation Review

## Data Availability

The #BeeWell dataset used in the current study is available via protected access arrangements at the University of Manchester (UoM). The FBB dataset is subject to a data sharing agreement (DSA) between UoM and FBB and cannot be shared directly by the authors. Researchers interested in replicating the findings of the study will need to initiate a DSA with FBB. *
