# Supplementary material for: Preliminary Evaluation of a Targeted, School-Based Social and Emotional Learning Intervention for at Risk Youth: Football Beyond Borders
Source: Eval Rev. 2025 Mar 24;49(5):914–30. doi: 10.1177/0193841X251329459 (PMC12379023; doi:10.1177/0193841X251329459)
Supplement: Supplemental Material - Preliminary Evaluation of a Targeted, School-Based Social and Emotional Learning Intervention for at Risk Youth: Football Beyond Borders [file sj-pdf-1-erx-10.1177_0193841X251329459.pdf]

Preliminary Evaluation of a Targeted, School-Based Social and Emotional Learning Intervention for At Risk Youth: Football Beyond Borders: Supplemental Material

Below we document the propensity score matching (PSM) (Table S1), multiple imputation (MI) (Table S2 to S4 and Figure S1), placebo analyses (Tables S5 to S8), and alternative matching methods (Tables S9) used in the current study. In addition, we present results for analyses using transformed SWEMWBS scores (Figures S2, S3 and Tables S10 to S12), z-transformed SWEMWBS scores and alternative DiD estimator.

PSM and covariates balance

As stated in the main manuscript, to estimate the propensity score, a binary treatment assignment variable (1 = FBB, 0 = Not in FBB) is regressed on the 11 covariates in a logistic regression using a logit link function. Most of the covariates in the regression are binary variables (See Table S1, column 2). We commenced by matching each FBB child with one #Beewell child (Ratio = 1:1) without replacement based on their estimated propensity scores (PS). We use a caliper width of 0.2 of the standard deviation of the PS logit when matching (Austin, 2014). After matching, the Standardized Mean Difference (SMD), Kolmogorov-Smirnov (KS) statistics and the overlapping coefficient (OVL) were used as balance checking metrics (Table S1). The *cobalt* package for R was used to obtain those statistics (Greifer, 2023).

Table S1

SMD, KS and OVL Statistics Before and After PSM

		Unmatched			Matched		
		SMD	KS	OVL	SMD	KS	OVL
Distance	Distance	0.661	0.853	0.463	-0.001	0.026	0.000
Is Female	Binary	-0.280	0.280	0.280	0.000	0.000	0.000
Is FSM	Binary	0.286	0.286	0.286	-0.026	0.026	0.026
Is SEN	Binary	0.225	0.225	0.225	0.000	0.000	0.000
SWEMWBS Time 0	Contin.	-0.079	0.105	0.099	0.020	0.026	0.033
Is Year 7	Binary	0.065	0.065	0.065	0.000	0.000	0.000
Is Year 8	Binary	-0.230	0.230	0.230	0.000	0.000	0.000
Is Year 9	Binary	0.164	0.164	0.164	0.000	0.000	0.000
Is top Attainment	Binary	-0.189	0.189	0.189	0.000	0.000	0.000
Is bottom Attainment	Binary	0.560	0.560	0.560	0.000	0.000	0.000
Is top Adversity	Binary	0.659	0.659	0.659	0.000	0.000	0.000
Is bottom Adversity	Binary	-0.334	0.334	0.334	0.000	0.000	0.000

Note. SWEMWBS = Short Warwick Edinburgh Mental Wellbeing Scale score. SEN = Special educational needs. FSM = Free school meals eligibility.

Missingness and Multiple imputation

We used complete case analysis in the main manuscript. To ensure the robustness of our findings and account for potential biases introduced by missing data, we conducted a sensitivity analysis using multiple imputation. As in the main manuscript, we only included the complete case subsample of the #Beewell dataset, but for the FBB data (N = 54), we also included incomplete observations. In the FBB data, FSM and SEN were reported by the school and we found that the missingness was due to the reasons at the school level (Table S2). The results from the logistic regression suggest that school ID is the only significant predictor of missingness for FSM and SEN ($ps < 0.01$), which account for most of the missing values. Other variables are not significant predictors of the missingness for all the variables (all $p > 0.05$). It can be said that the missingness of the FSM and the SEN in the FBB data was not associated with the outcome variables, supporting the assumption of missing at random or missing completely at random. Following the linking of these two datasets, we applied multiple imputation,

generating 50 datasets for subsequent analysis. The imputation process utilized the *Mice* package in R, employing the *random forest* method.

Table S2

Missingness in the treated sample (At Risk)

Variable	Observed N	Missing N	Missing Percent (%)
FSM	49	5	9.3
Year Group	54	0	0
Gender	54	0	0
SEN	50	4	7.4
FBB	54	0	0
SWEMWBS (Pre)	52	2	3.7
SWEMWBS (Post)	53	1	1.9

Note. SWEMWBS = Short Warwick Edinburgh Mental Wellbeing Scale score. SEN = Special educational needs. FSM = Free school meals eligibility.

The multiple imputation regression analysis revealed a statistically significant positive effect of FBB on SWEMWBS scores, with an ATT of 2.40, p = 0.02, 95%CI = (0.37, 4.43) (see Table S3 for estimated results, and Table S4 and Figure S1 for covariates balance checks). Importantly, the results obtained through multiple imputation align with those from the complete case analysis, reinforcing the robustness and reliability of our reported findings.

Table S3

The Impact of FBB on Mental Wellbeing (SWEMWBS) Estimated from Multiple Imputation

	Model 1	Model 2
$ATT\ D_{i,t}$	2.40<br>(0.37, 4.43)	2.44<br>(0.40, 4.48)
Time FE ϕ_t	Yes	Yes
Individual FE α_i	Yes	Yes
$X_i \times Post_t$	No	Yes

Note. The table reports the DiD effect resulting from employing the two-way fixed effects (TWFE) regressions with *fixest* package for R (Berge L, 2018).

Both models included the time fixed effect and individual fixed effect. In model 2 we controlled for the interaction between post treatment period and covariates, $X_i \times Post_t$. The covariates include SEN, Gender, FSM, Year 8, Year 9. Year 7, top attainment, bottom attainment, top adversity and bottom adversity indicators were eventually dropped as there was no variance in these variables in the matched sample (see Table 1).

The 95% confidential intervals of the estimate are shown in parentheses.

SWEMWBS = Short Warwick Edinburgh Mental Wellbeing Scale score. SEN = Special educational needs. FSM = Free school meals eligibility. AR = At risk. ATT = Average treatment effect on the treated (over the region of common support). $D_{i,t}$ is the indicator of treatment status of individual i previous to time *t*.

Table S4

Group Characteristics Before and After PSM for Multiple Imputations

		Unmatched				Matched			
		FBB = 0 (N = 8015)	FBB_AR=1 (N = 54)	p value	SMD	FBB = 0 (N = 41)	FBB_AR=1 (N =41)	p value	SMD
SWEMWBS T1		23.43 (5.82)	23.17 (5.49)	0.745	0.046	21.80 (6.40)	23.78 (5.31)	0.132	0.336
SWEMWBS T0		23.61 (5.69)	22.96 (5.55)	0.411	0.116	23.66 (5.55)	23.49 (5.67)	0.891	0.03
Is SEN (%)	0	7031 (87.7)	33 (66.0)	<0.001	0.533	25 (61.0)	26 (63.4)	1	0.05
	1	984 (12.3)	17 (34.0)			16 (39.0)	15 (36.6)		
Is Female (%)	0	4027 (50.2)	44 (81.5)	<0.001	0.698	35 (85.4)	34 (82.9)	1	0.067
	1	3988 (49.8)	10 (18.5)			6 (14.6)	7 (17.1)		
Is FSM (%)	0	6303 (78.6)	24 (49.0)	<0.001	0.649	22 (53.7)	23 (56.1)	1	0.049
	1	1712 (21.4)	25 (51.0)			19 (46.3)	18 (43.9)		
Is Year 7 (%)	0	8015 (100.0)	48 (88.9)	<0.001	0.5	41 (100.0)	41 (100.0)	NA	<0.001
	1	0 (0.0)	6 (11.1)			0(0.0)	0(0.0)		
Is Year 8 (%)	0	76 (0.9)	15 (27.8)	<0.001	0.828	1 (2.4)	2 (4.9)	1	0.13
	1	7939 (99.1)	39 (72.2)			40 (97.6)	39 (95.1)		
Is Year 9 (%)	0	7939 (99.1)	45 (83.3)	<0.001	0.577	40 (97.6)	39 (95.1)	1	0.13
	1	76 (0.9)	9 (16.7)			1 (2.4)	2 (4.9)		
Is top attainment (%)	0	6500 (81.1)	54 (100.0)	0.001	0.683	41 (100.0)	41 (100.0)	NA	<0.001
	1	1515 (18.9)	0 (0.0)			0(0.0)	0(0.0)		
Is bottom attainment (%)	0	4488 (56.0)	0 (0.0)	<0.001	1.595	0(0.0)	0(0.0)	NA	<0.001
	1	3527 (44.0)	54 (100.0)			41 (100.0)	41 (100.0)		
Is top adversity (%)	0	5280 (65.9)	0 (0.0)	<0.001	1.965	0(0.0)	0(0.0)	NA	<0.001
	1	2735 (34.1)	54 (100.0)			41 (100.0)	41 (100.0)		
Is bottom adversity (%)	0	5336 (66.6)	54 (100.0)	<0.001	1.002	41 (100.0)	41 (100.0)	NA	<0.001
	1	2679 (33.4)	0 (0.0)			0(0.0)	0(0.0)		

Note. For binary variables, p-values are obtained from Pearson's chi-squared test with continuity correction and SMD (standardized mean difference) is the multivariate Mahalanobis distance between group-specific proportions. For continuous variables, p-values are obtained from one-way ANOVA assuming equal variance.

The statistics are obtained using the R package *tableone*. SWEMWBS = Short Warwick Edinburgh Mental Wellbeing Scale score. SEN = Special educational needs. FSM = Free school meals eligibility. AR = At Risk.

Figure S1

Covariate Balance Before and After PSM for Multiple Imputations

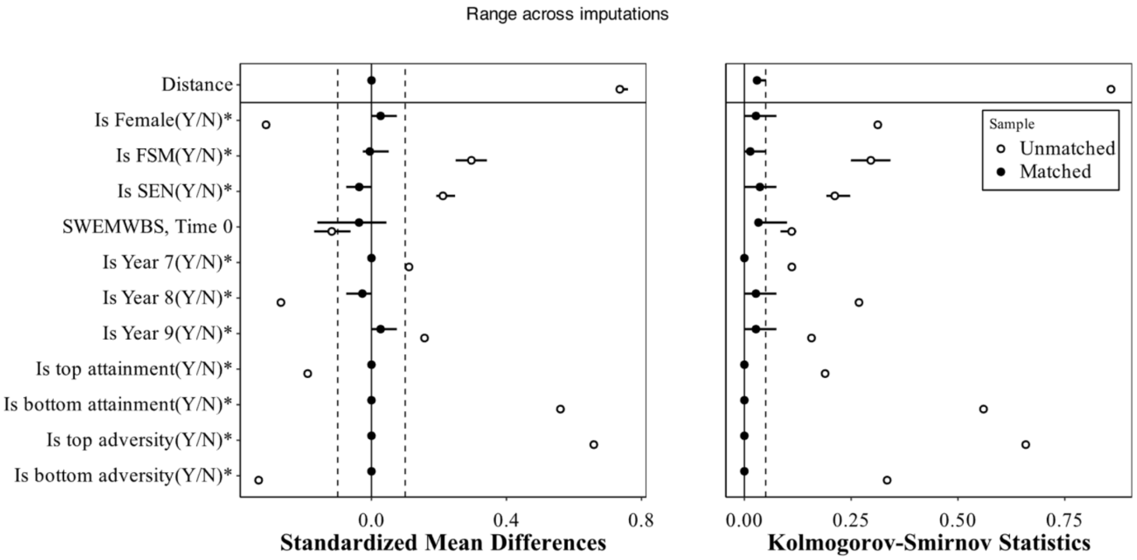

Note. SWEMWBS = Short Warwick Edinburgh Mental Wellbeing Scale score. SEN = Special educational needs. FSM = Free school meals eligibility.

PSM and DiD results for Passive learner

We applied the same analysis strategy to the passive learners. The effect of FBB on the SWEMWBS scores in the passive learners is positive but not statistically significant, at about 0.36 points (95% CI = -1.50 to 2.21), equivalent to d = 0.07. This effect is stable after we control for the interaction between the post intervention period (time) and the covariates (Table S5). The group characteristics before and after propensity score matching are shown in Table S6.

Table S5

The Impact of FBB on Mental Wellbeing (SWEMWBS) of Passive Learners

	Model 1	Model 2
ATT $D_{i,t}$	0.36<br>(-1.50, 2.21)	0.39<br>(-1.48, 2.26)
Time FE ϕ_t	Yes	Yes
Individual FE α_i	Yes	Yes
$X_i \times Post_t$	No	Yes

Note. The table reports the DiD effect resulting from employing the two-way fixed effects (TWFE) regressions with *fixest* package for R (Berge L, 2018).

Both models included the time fixed effect and individual fixed effect. In model 2 we controlled for the interaction between post treatment period and covariates, $X_i \times Post_t$. The covariates include SEN, Gender, FSM, Year 8, Year 9. Year 7, top attainment, bottom attainment, top adversity and bottom adversity indicators were eventually dropped as there was no variance in these variables in the matched sample (see Table 1).

The 95% confidential intervals of the estimate are shown in parentheses.

SWEMWBS = Short Warwick Edinburgh Mental Wellbeing Scale score. SEN = Special educational needs. FSM = Free school meals eligibility. AR = At risk. ATT = Average treatment effect on the treated (over the region of common support). $D_{i,t}$ is the indicator of treatment status of individual i previous to time *t.*

Table S6

Group Characteristics Before and After Propensity Score Matching (Passive Learner)

		Unmatched				Matched			
		FBB = 0<br>(N = 8015)	FBB_PL=1<br>(N = 72)	p value	SMD	FBB = 0<br>(N = 59)	FBB_PL=1<br>(N =59)	p value	SMD
SWEMWBS T1		23.43 (5.82)	23.97 (5.62)	0.432	0.095	23.49 (6.36)	23.98 (5.60)	0.657	0.082
SWEMWBS T0		23.61 (5.69)	24.58 (5.19)	0.149	0.178	24.34 (4.33)	24.47 (4.59)	0.869	0.03
Is SEN (%)	0	7031 (87.7)	54 (75.0)	0.002	0.331	43 (72.9)	44 (74.6)	1	0.039
	1	984 (12.3)	18 (25.0)			16 (27.1)	15 (25.4)		
Is Female (%)	0	4027 (50.2)	61 (84.7)	<0.001	0.792	48 (81.4)	48 (81.4)	1	<0.001
	1	3988 (49.8)	11 (15.3)			11 (18.6)	11 (18.6)		
Is FSM (%)	0	6303 (78.6)	41 (56.9)	<0.001	0.477	33 (55.9)	33 (55.9)	0.846	<0.001
	1	1712 (21.4)	31 (43.1)			26 (44.1)	26 (44.1)		
Is Year 7 (%)	0	8015 (100.0)	63 (87.5)	<0.001	0.535	59 (100.0)	59 (100.0)	NA	<0.001
	1	0 (0.0)	9 (12.5)			0(0.0)	0(0.0)		
Is Year 8 (%)	0	76 (0.9)	25 (34.7)	<0.001	0.983	12 (20.3)	12 (20.3)	0.74	<0.001
	1	7939 (99.1)	47 (65.3)			47 (79.7)	47 (79.7)		

Is Year 9 (%)	0	7939 (99.1)	56 (77.8)	<0.001	0.705	47 (79.7)	47 (79.7)	0.74	<0.001
	1	76 (0.9)	16 (22.2)			12 (20.3)	12 (20.3)		
Is top attainment (%)	0	6500 (81.1)	72 (100.0)	<0.001	0.683	59 (100.0)	59 (100.0)	NA	<0.001
	1	1515 (18.9)	0 (0.0)			0(0.0)	0(0.0)		
Is bottom adversity (%)	0	5280 (65.9)	72 (100.0)	<0.001	1.965	59(100.0)	59(100.0)	NA	<0.001
	1	2735 (34.1)	0 (0.0)			0 (0.0)	0 (0.0)		

Note. For binary variables, p-values are obtained from Pearson's chi-squared test with continuity correction and SMD (standardized mean difference) is the multivariate Mahalanobis distance between group-specific proportions. For continuous variables, p-values are obtained from one-way ANOVA assuming equal variance. The statistics are obtained using the R package *tableone*. SWEMWBS = Short Warwick Edinburgh Mental Wellbeing Scale score. SEN = Special educational needs. FSM = Free school meals eligibility. PL = Passive Learner.

PSM and DiD results for Role Models

We applied the same analysis strategy to the role models. The effect of FBB on the SWEMWBS scores in role models is positive but not statistically significant, at about 0.39 points (95% CI = -2.76 to 3.54), equivalent to *d* = 0.09 (Table S7). The group characteristics before and after propensity score matching are shown in Table S8.

Table S7
The Impact of FBB on Mental Wellbeing (SWEMWBS) of Role Models

	Model 1	Model 2
$ATT\ D_{i,t}$	0.39<br>(-2.76, 3.54)	0.27<br>(-2.94, 3.48)
Time FE ϕ_t	Yes	Yes
Individual FE α_i	Yes	Yes
$X_i \times Post_t$	No	Yes

Note. The table reports the DiD effect resulting from employing the two-way fixed effects (TWFE) regressions with *fixest* package for R (Berge L, 2018).

Both models included the time fixed effect and individual fixed effect. In model 2 we controlled for the interaction between post treatment period and covariates, $X_i \times Post_t$. The covariates include SEN, Gender, FSM, Year 8, Year 9. Year 7, top attainment, bottom attainment, top adversity and bottom adversity indicators were eventually dropped as there was no variance in these variables in the matched sample (see Table 1).

The 95% confidential intervals of the estimate are shown in parentheses.

SWEMWBS = Short Warwick Edinburgh Mental Wellbeing Scale score. SEN = Special educational needs. FSM = Free school meals eligibility. ATT = Average treatment effect on the treated (over the region of common support). $D_{i,t}$ is the indicator of treatment status of individual i previous to time *t*.

Table S8

Group Characteristics Before and After Propensity Score Matching (Role Models)

		Unmatched				Matched			
		FBB = 0 (N = 8015)	FBB_RM=1 (N = 35)	p value	SMD	FBB = 0 (N = 18)	FBB_RM=1 (N = 18)	p value	SMD
SWEMWBS T1		23.43 (5.82)	24.94 (4.75)	0.125	0.285	25.50 (4.94)	25.22 (3.75)	0.85	0.063
SWEMWBS T0		23.61 (5.69)	24.51 (4.81)	0.349	0.171	25.00 (4.37)	24.33 (4.31)	0.648	0.154
Is SEN (%)	0	7031 (87.7)	31 (88.6)	1	0.026	16 (88.9)	16 (88.9)	1	<0.001
	1	984 (12.3)	4 (11.4)			2 (11.1)	2 (11.1)		
Is Female (%)	0	4027 (50.2)	30 (85.7)	<0.001	0.822	17 (94.4)	16 (88.9)	1	0.202
	1	3988 (49.8)	5 (14.3)			1 (5.6)	2 (11.1)		
Is FSM (%)	0	6303 (78.6)	26 (74.3)	0.674	0.103	18 (100.0)	18 (100.0)	NA	<0.001
	1	1712 (21.4)	9 (25.7)			0(0.0)	0(0.0)		
Is Year 7 (%)	0	8015 (100.0)	33 (94.3)	<0.001	0.348	18 (100.0)	18 (100.0)	NA	<0.001
	1	0 (0.0)	2 (5.7)			0(0.0)	0(0.0)		
Is Year 8 (%)	0	76 (0.9)	11 (31.4)	<0.001	0.909	2 (11.1)	2 (11.1)	1	<0.001
	1	7939 (99.1)	24 (68.6)			16 (88.9)	16 (88.9)		
Is Year 9 (%)	0	7939 (99.1)	26 (74.3)	<0.001	0.782	16 (88.9)	16 (88.9)	1	<0.001
	1	76 (0.9)	9 (25.7)			2 (11.1)	2 (11.1)		
Is top attainment (%)	0	6500 (81.1)	0 (0.0)	<0.001	2.929	0(0.0)	0(0.0)	NA	<0.001
	1	1515 (18.9)	35 (100.0)			18 (100.0)	18 (100.0)		
Is bottom attainment (%)	0	4488 (56.0)	35 (100.0)	<0.001	1.254	18 (100.0)	18 (100.0)	NA	<0.001
	1	3527 (44.0)	0 (0.0)			0(0.0)	0(0.0)		
Is top adversity (%)	0	5280 (65.9)	35 (100.0)	<0.001	1.018	18 (100.0)	18 (100.0)	NA	<0.001
	1	2735 (34.1)	0 (0.0)			0(0.0)	0(0.0)		
Is bottom adversity (%)	0	5336 (66.6)	0 (0.0)	<0.001	1.996	0(0.0)	0(0.0)	NA	<0.001
	1	2679 (33.4)	35 (100.0)			18 (100.0)	18 (100.0)		

Note. For binary variables, p-values are obtained from Pearson's chi-squared test with continuity correction and SMD (standardized mean difference) is the multivariate Mahalanobis distance between group-specific proportions. For continuous variables, p-values are obtained from one-way ANOVA assuming equal variance. The statistics are obtained using the R package *tableone*. SWEMWBS = Short Warwick Edinburgh Mental Wellbeing Scale score. SEN = Special educational needs. FSM = Free school meals eligibility. RM = Role Models.

Alternative Matching Approaches

Here we provided results from other alternative matching approaches (Table S9). The results from seven out of the nine alternative matching methods show significant average treatment effects on the treated. However, the remaining two matching methods (3-nearest neighbours and optimal matching) gave insignificant results.

Table S9

Results from Alternative Matching Approaches

Sample	*ATT*[a]	*d*	*95% CI*[b]	*p*	*N*	*balanced?*
1-NN[c] without caliper	2.52	0.45	0.64, 4.41	0.01	46+46	N
1-NN with replacement	2.51	0.45	0.32, 4.71	0.03	38+35	Y
3-NN	1.49	0.31	-0.003, 2.99	0.0504	38+107	Y
6-NN	1.50	0.31	0.01, 2.99	0.048	38+195	Y
10-NN	1.46	0.26	0.02, 2.91	0.047	38+295	Y
Optimal	1.17	0.20	-0.69, 3.04	0.21	46+46	N
Full	1.48	0.26	0.15, 2.83	0.03	38+8003	N
Exact	1.68	0.32	0.04, 3.32	0.04	35+288	Y
Genetic[d]	1.97	0.36	0.01, 3.94	0.049	38+38	Y

Note. [a] The average treatment effect on the treated (*ATT*) were obtained using the two-way fixed effects (TWFE) regressions with the *fixest* package for R. The model specification is the same as Model 1 in Table 2 of the manuscript.

[b] The 95% CI was calculated based on the cluster-robust SEs for matches without replacement and based on the HC1 SEs for matches with replacement.

[c] NN = Nearest Neighbour Matching.

[d] The results from the genetic matching were subject to the randomiser seeds.

Transformed SWEMWBS scores

When transformed SWEMWBS scores (Stewart-Brown et al, 2009) were used, the results and effect size in the main analysis remained the same, with b = 2.01, 95% CI = (0.27, 3.75), and effect size d = 0.44, see Table S10. With transformed scores, the matched sample was reduced from 38 pairs to 37 pairs (Table S12). The covariates are very similar in the treated and control groups, see Figures S2 & S3 and Table S12.

Using the multiple imputation method, regression analysis revealed a statistically significant positive effect of FBB on transformed SWEMWBS scores, with an estimate of 1.86 (0.19, 3.52), see Table S11.

Table S10

The Impact of FBB on Mental Wellbeing (Transformed SWEMWBS)

	Model 1	Model 2
$ATT\ D_{i,t}$	2.01<br>(0.27, 3.75)	2.06<br>(0.28, 3.85)
Time FE ϕ_t	Yes	Yes
Individual FE α_i	Yes	Yes
$X_i \times Post_t$	No	Yes

Table S11

The Impact of FBB on Mental Wellbeing (Transformed SWEMWBS) Estimated from Multiple Imputation

	Model 1	Model 4
$ATT\ D_{i,t}$	1.86<br>(0.19, 3.52)	1.89<br>(0.21, 3.58)
Time FE ϕ_t	Yes	Yes
Individual FE α_i	Yes	Yes
$X_i \times Post_t$	No	Yes

Figure S2

Covariate Balance Before and After PSM (Transformed SWEMWBS Scores)

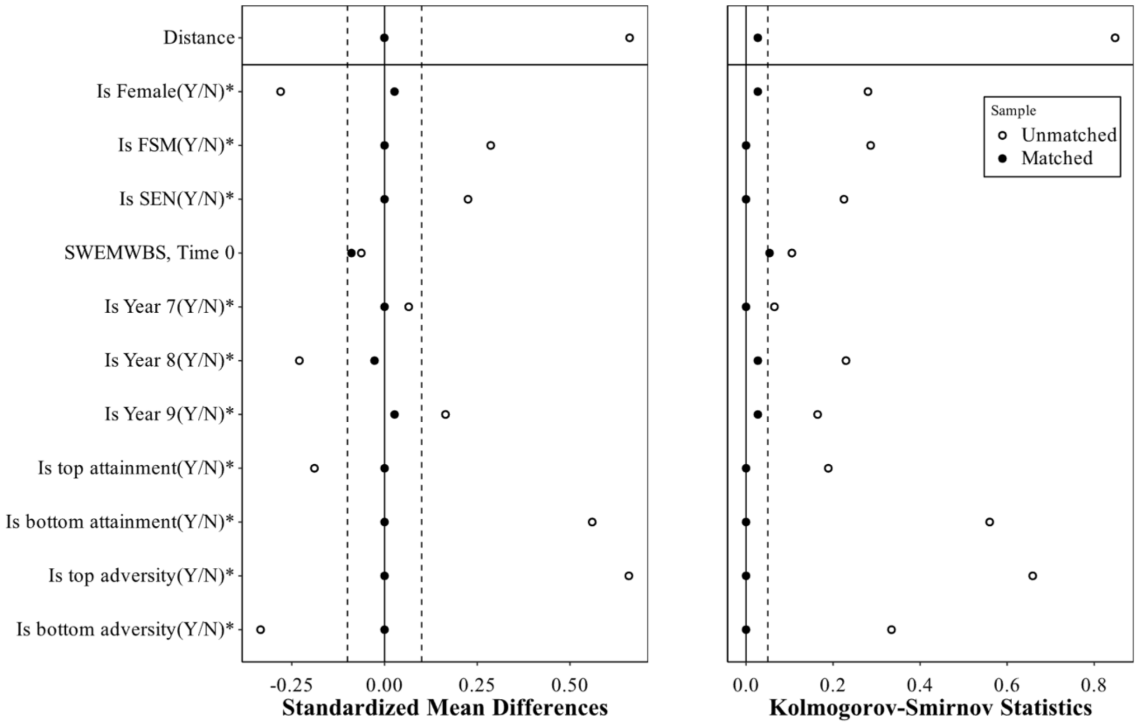

Figure S3

Jitter Plot Demonstrating the Distribution of Propensity Scores in Matched and Unmatched Samples (Transformed SWEMWBS Scores)

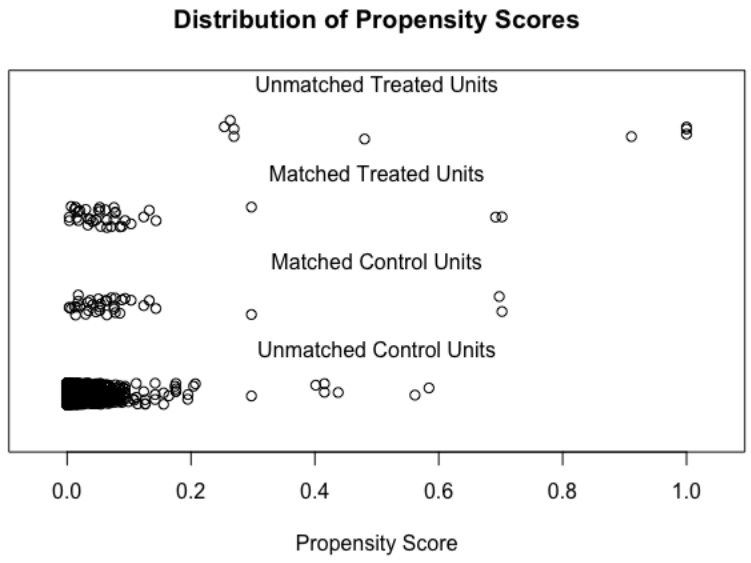

Table S12

Group Characteristics Before and After Propensity Score Matching

		Unmatched				Matched			
		FBB = 0 (N = 8015)	FBB_AR=1 (N =46)	p value	SMD	FBB = 0 (N = 37)	FBB_AR=1 (N =37)	p value	SMD
SWEMWBS T1		21.71 (4.99)	21.44 (4.38)	0.723	0.056	19.77 (5.16)	21.36 (3.95)	0.141	0.346
SWEMWBS T0		21.83 (4.79)	21.54 (4.68)	0.679	0.062	21.80 (4.55)	21.39 (4.40)	0.689	0.093
Is SEN (%)	0	7031 (87.7)	30 (65.2)	<0.001	0.55	22 (59.5)	22 (59.5)	1	<0.001
	1	984 (12.3)	16 (34.8)			15 (40.5)	15 (40.5)		
Is Female (%)	0	4027 (50.2)	36 (78.3)	<0.001	0.611	31 (83.8)	30 (81.1)	1	0.071
	1	3988 (49.8)	10 (21.7)			6 (16.2)	7 (18.9)		
Is FSM (%)	0	6303 (78.6)	23 (50.0)	<0.001	0.626	21 (56.8)	21 (56.8)	1	<0.001
	1	1712 (21.4)	23 (50.0)			16 (43.2)	16 (43.2)		
Is Year 7 (%)	0	8015 (100.0)	43 (93.5)	<0.001	0.374	37 (100.0)	37 (100.0)	NA	<0.001
	1	0 (0.0)	3 (6.5)			0 (0.0)	0 (0.0)		
Is Year 8 (%)	0	76 (0.9)	11 (23.9)	<0.001	0.742	2 (5.4)	3 (8.1)	1	0.108
	1	7939 (99.1)	35 (76.1)			35 (94.6)	34 (91.9)		
Is Year 9 (%)	0	7939 (99.1)	38 (82.6)	<0.001	0.594	35 (94.6)	34 (91.9)	1	0.108
	1	76 (0.9)	8 (17.4)			2 (5.4)	3 (8.1)		
Is top attainment (%)	0	6500 (81.1)	46 (100.0)	0.002	0.683	37 (100.0)	37 (100.0)	NA	<0.001
	1	1515 (18.9)	0 (0.0)			0 (0.0)	0 (0.0)		
Is bottom attainment (%)	0	4488 (56.0)	0 (0.0)	<0.001	1.595	0 (0.0)	0 (0.0)	NA	<0.001
	1	3527 (44.0)	46 (100.0)			37 (100.0)	37 (100.0)		
Is top adversity (%)	0	5280 (65.9)	0 (0.0)	<0.001	1.965	0 (0.0)	0 (0.0)	NA	<0.001
	1	2735 (34.1)	46 (100.0)			37 (100.0)	37 (100.0)		
Is bottom adversity (%)	0	5336 (66.6)	46 (100.0)	<0.001	1.002	37 (100.0)	37 (100.0)	NA	<0.001
	1	2679 (33.4)	0 (0.0)			0 (0.0)	0 (0.0)		

Note. For binary variables, p-values are obtained from Pearson's chi-squared test with continuity correction and SMD is the multivariate Mahalanobis distance between group-specific proportions. For continuous variables, p-values are obtained from one-way ANOVA assuming equal variance. The statistics are obtained using the R package *tableone*.

z-transformed SWEMWBS scores (z-score)

Although the Shapiro-Wilk normality test indicated that we couldn't reject the null hypothesis of normality of the dependent variable in the four groups (with all *ps* > .14, see also Figure S4), a sensitivity analysis was

performed using the z-transformed SWEMWBS score to ensure robustness. When using the z-transformed SWEMWBS score, the matched sample obtained was the same as the matched sample used in the main analysis. As a result, the estimated effect size remained the same.

Figure S4

The Distribution of the Raw SWEMWBS scores Across the Four Groups.

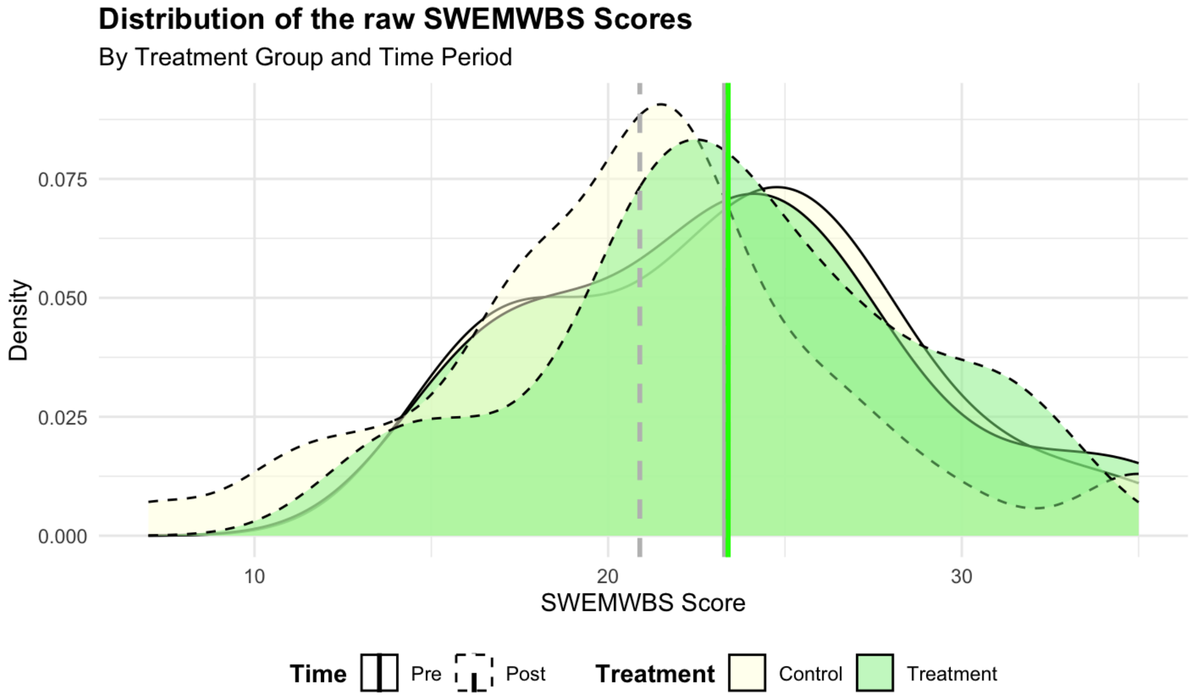

Results from alternative estimator

The Double Robust DiD estimator (Sant'Anna & Zhao, 2020) was applied to the matched data used in the main analysis. The results of using this estimator also suggested a significant effect of FBB on SWEMWBS scores, with ATT = 2.37, se = 0.96, t = 2.47, p = 0.01, 95%CI = [0.49, 4.25].

Supplementary Material References

Arel-Bundock V (2024). _marginaleffects: Predictions, comparisons, slopes, marginal means, and hypothesis tests_. *R package version 0.17.0.9002*, accessed at https://marginaleffects.com/ (January 2024).

Austin, P.C. (2014), A comparison of 12 algorithms for matching on the propensity score. *Statistical Medicine*, 33: 1057-1069. https://doi.org/10.1002/sim.6004

Berge L (2018). "Efficient estimation of maximum likelihood models with multiple fixed-effects: the R package FENmlm." *CREA Discussion Papers.*

Greifer, N. (2023). cobalt: Covariate balance tables and plots. *R package version 4.5.2*.

Sant'Anna, P. H. C., & Zhao, J. (2020). Doubly robust difference-in-differences estimators. *Journal of Econometrics*, *219*(1), 101-122. https://doi.org/10.1016/j.jeconom.2020.06.003

Stewart-Brown, S., Tennant, A., Tennant, R. et al. Internal construct validity of the Warwick-Edinburgh Mental Well-being Scale (WEMWBS): a Rasch analysis using data from the Scottish Health Education Population Survey. *Health Qual Life Outcomes* 7, 15 (2009). https://doi.org/10.1186/1477-7525-7-15
